# Methylxanthines induce structural and functional alterations of the cardiac system in zebrafish embryos

**DOI:** 10.1186/s40360-017-0179-9

**Published:** 2017-11-15

**Authors:** Ram Manohar Basnet, Daniela Zizioli, Michela Guarienti, Dario Finazzi, Maurizio Memo

**Affiliations:** 1 Department of Molecular and Translational Medicine, University of Brescia, Viale Europa, 11, 25123 Brescia, Italy; 2 Clinical Chemistry Laboratory, ASST Spedali Civili di Brescia, 25123 Brescia, Italy

**Keywords:** Embryotoxicity, Teratogenicity, Methylxanthines, Heart rate, Cardiac dysfunction

## Abstract

**Background:**

Zebrafish embryos are emerging as a model for pharmacological and toxicological studies. We used zebrafish embryos to study the general toxicity and cardiovascular effects of eight methylxanthines: aminophylline, caffeine, diprophylline, doxofylline, etophylline, 3-isobutyl-1-methylxanthine (IBMX), pentoxifylline and theophylline.

**Methods:**

Microinjections of the eight methylxanthines were performed in 1-2 cell stage zebrafish embryos and the general toxicity and cardiovascular effects were analyzed at different time points. Embryotoxicity and teratogenicity were evaluated to understand the general toxicity of these compounds. Structural and functional alterations of the heart were evaluated to assess the cardiovascular effects.

**Results:**

Our results showed different activity patterns of the methylxanthines drugs. Caffeine, IBMX, pentoxifylline and theophylline were highly embryotoxic and teratogenic; aminophylline, doxofylline and etophylline were embryotoxic and teratogenic only at higher doses, and diprophylline showed a minimal (<10%) embryotoxicity and teratogenicity. Most of these drugs induced structural alteration of the heart in 20-40% of the injected embryos with the maximum dose. This structural alteration was fatal with the embryos ultimately dying within 120 hpf. All the drugs induced a transient increase in heart rate at 48 hpf which returned to baseline within 96 hpf. This functional effect of methylxanthines showed similarity to the studies done in humans and other vertebrates.

**Conclusion:**

Our results indicate the potential toxicity and teratogenicity of different methylxanthines in the embryos during embryonic development, the most sensitive period of life. Although interspecies differences need to be considered before drawing any conclusion, our study elucidated that a single exposure of methylxanthines at therapeutic range could induce cardiac dysfunction besides causing embryotoxicity and teratogenicity. Of all the drugs, diprophylline appeared to be safer, with lower degree of embryotoxicity, teratogenicity and cardiac toxicity as compared to other methylxanthines.

**Electronic supplementary material:**

The online version of this article (10.1186/s40360-017-0179-9) contains supplementary material, which is available to authorized users.

## Background

Methylxanthines are one of the widely consumed substances in the world [1, 2]. The sources of methylxanthines include tea, coffee, chocolate, energy drinks and drugs [1]. They can be naturally occurring, such as caffeine, theobromine and theophylline, or synthetic derivatives, such as diprophylline and pentoxifylline [3, 4]. Beverages rich in methylxanthines have been consumed in different cultures across centuries [1, 5, 6]. These substances are also used for the treatment of diverse medical conditions such as asthma, chronic obstructive pulmonary disease (COPD), peripheral vascular disease, obesity, hyperlipidemia and apnea of prematurity [3, 7–13]. Therefore, these compounds are regularly ingested in a daily basis through various beverages and drugs. All these drugs have similar mechanism of action; they act via inhibition of phosphodiesterase enzyme, antagonism of  adenosine and GABA_A_ receptors, and blocking of the calcium channels [1, 3, 5].

The human exposure to methylxanthines begins as early as gestation and continues throughout the life [14]. Although in human, fatal cases of methylxanthine toxicity with theophylline, aminophylline [15], caffeine [16, 17] and pentoxifylline [18] from accidental overdose and suicide have been reported, they are rare and mostly iatrogenic [14]. Rather, the therapeutic use of methylxanthines has been limited by their life threatening adverse effects, such as cardiac arrhythmias and intractable seizures [19]. For instance, theophylline has been replaced to fourth line of therapy in the treatment of chronic obstructive pulmonary disease as it causes arrhythmia and seizures. Also other methylxanthines, like aminophylline and caffeine, have been implicated in several types of cardiac arrhythmia. As cardiotoxicity is one of the principal reasons for the drug withdrawal [20], prior information of the cardiotoxic property of these drugs could save time and money in the drug development process.

Cardiac toxicity and hemodynamic perturbations are two major aspects of methylxanthine toxicity [21]. Tachydysrhythmias which includes sinus tachycardia and premature ventricular contraction are the most common dysrhythmia observed with methylxanthines toxicity [22]. They also cause widened pulse pressure, hypotension, myocardial ischemia and infarction [23]. The complexity and relevance of the observed cardiac toxicity strengthen the importance of further studies of methylxanthines induced cardiotoxicity.

The purpose of our study was twofold. On one hand, we wanted to study the general and cardiac toxicity of methylxanthines. At the same time, we also wanted to study the teratogenicity of these drugs. To achieve our goals, we employed zebrafish embryos which might provide us the insight regarding the toxicity and teratogenicity of these drugs simultaneously.

Zebrafish are emerging as a predictive animal model for assessing the in vivo effects and toxicity of various compounds and drugs [24–27] with numerous studies showing overwhelming similarity between mammalian and zebrafish toxicity [20, 24, 27, 28]. Moreover, recent evidences have also shown a high degree of similarity between the pharmacological responses of zebrafish and human to known cardiotoxins [20]. In addition to the general advantages over other animal models such as transparent embryos, external fertilization, high fecundity, economical and easy manipulation [29], zebrafish embryos have features which favor its use in the study of drug induced cardiovascular effects. They have a simple cardiac and vascular system, and the molecular mechanisms underlying vascular tree development strongly resembles those of higher vertebrates, showing a high degree of anatomical and functional conservation [30]. At 48 hpf, the zebrafish cardiovascular system is fully functional with a complex repertoire of ion channels and metabolic processes already developed [31]. Like mammals, they possess a pacemaker which controls heart beat, the blood is collected by the atrium and is pumped throughout the body by the ventricle. The transparency of zebrafish embryos makes it easy to study the heart rate and rhythm, cardiac morphology and blood circulation [31, 32]. Furthermore, the availability of transgenic embryos has made it easy to study the in vivo effects of cardiovascular system effectively.

In this study, we investigated the general and cardiac toxicity of eight methylxanthines compounds: aminophylline, caffeine, diprophylline, doxofylline, etophylline, 3-isobutyl-1-methylxanthine (IBMX), pentoxifylline and theophylline in zebrafish embryos. These methylxanthines were selected based on their presence in commonly consumed beverages such as coffee, tea and mate, therapeutic uses in diverse medical conditions such as COPD, peripheral vascular disorders, hyperlipidemia and apnoea of prematurity and their water solubility. Zebrafish embryos were microinjected with each methylxanthine compound with five pre-determined graded doses of each drug into the yolk of 1-2 cell zebrafish embryos. We firstly studied the general toxicity caused by these drugs in zebrafish embryos. Cumulative mortality induced by these drugs was calculated after 72 hpf. The phenotype of surviving/alive embryos was classified as normal, mild and severe based on the observance of morphological defects. Secondly, we studied the cardiac toxicity of methylxanthines taking into account both the structural and functional aspects of the heart. Our study aims to provide the insight into the cardiac teratogenicity within the realm of general and cardiac toxicity of methylxanthines.

## Methods

### Drugs

Eight methylxanthine drugs were used in the study: aminophylline, caffeine, diprophylline, etophylline, IBMX, theophylline from Sigma and doxofylline and pentoxifylline from Abcam. Metoprolol, a β-blocker used for the cardiovascular study was obtained from Sigma.

### Zebrafish maintenance and collection of eggs

Zebrafish were maintained and used in accordance with the Italian and European rules on animal use following protocols approved by the local Committee (OPBA protocol nr 211B5.24) and authorized by the Ministry of Health (authorization number 393/2017-PR).

Adult AB wild type and transgenic line tg (*Bmp:EGFP*) [33] zebrafish were raised and maintained in 14: 10 h light: dark cycle at 28 °C in a circulating system maintained at pH 7.0 and conductivity between 400 and 500 μs. Fishes were fed three time per day with a combination of granular food (from special diet services, SDS, Witham, UK) in the morning and evening and artemia freshly prepared in the laboratory in the afternoon (cysts bought from SDS, Witham, UK).

Adult zebrafish were used for egg production. Male and female were put in the breeding tank overnight and next morning fresh spawned embryos were collected. Any unfertilized eggs, dead or bad embryos were removed and the good quality embryos at 1-2 cell stage were selected for microinjection.

### Microinjection

Zebrafish embryos were injected with five different concentrations of eight methylxanthines at 1-2 cell stage. The total volume of injection was 5 nL/embryo and a minimum of 100 embryos were injected for each concentration. Each compound was co-injected with 0.05% phenol red as a tracer. The total quantity of drug injected per embryo was obtained by calculating the total amount of drug in nanogram (ng) present in 5 nL of all the five injected concentrations of each drug. The doses of the drugs injected per embryo ranged from 0.25 ng to 20 ng (Table 1). 3, 4-dichloroaniline (DCA) was used as positive control and embryos injected with 0.05% phenol red in sterile water acted as negative control. The non-injected embryos were used as a control to confirm that the needle pricking per se did not have any effects in the embryos. After microinjection, embryos were collected in Petri dish and maintained in fish water at 28 °C until further evaluation. Any defective or damaged embryos were discarded. The procedure was performed three times independently and the results are the mean ± S.D. of three experiments.

**Table 1:** Doses of methylxanthines injected per embryo in nanogram

Methylxanthines	Injected dose (ng/ embryo)				
Aminophylline	0.5	1.25	2.5*	3.75	5
Caffeine	0.25	0.5	0.75*	1.5	2.5
Diprophylline	1	2.5	5*	10	20
Doxofylline	1	2	3*	5	7.5
Etophylline	0.5	1	2*	3	5
IBMX	0.15	0.25	0.5*	0.75	1
Pentoxifylline	0.15	0.5	1*	2	3
Theophylline	0.25	0.5	1*	1.5	2.5
Dichloroaniline	0.015	0.05	0.1*		
Metoprolol	0.005	0.01	0.018*	0.037	0.075

The doses labeled with asterisk (*) were selected for the experiments in the transgenic tg (*Bmp:EGFP*) embryos to study the long term effect of methylxanthines in heart rate. Dichloroaniline was used as positive control in evaluation of mortality and general morphology, and  metoprolol was used  as internal control in evaluation of heart rate

### Evaluation of mortality and general morphology

Mortality of the injected embryos was recorded at each time points of 24, 48 and 72 hpf. The survival rates of each drugs was calculated and a dose-response survival graph was plotted at 72 hpf. A thorough evaluation of morphological endpoints from head to tail was performed at 48 and 72 hpf (Table 2). The phenoype of the embryos was then classified into normal, mild or severe based on the abnormality of evaluated endpoints. The morphological evaluation and phenotype grading was performed under direct visualization with Leica MZ 16F Stereomicroscope (Germany).

**Table 2:** Criteria for the grading of embryos into normal, mild and severe phenotypes at 48 hpf

Morphological endpoints	Normal	Mild	Severe
Head	Flexed	Straight	Extended
Tail	Properly detached and straight	Normal length and curved	Short length and curved
Anterior-posterior (AP) axis	Normal head and tail position	AP axis mildly disrupted	AP axis not well formed
Yolk	Transparent	Bigger than control and/or deformed	At least double than size of control with necrosis
Heart beat	Regular with synchronous contractions	Slow or fast	Slow or fast with asynchronous contractions
Pericardial edema	Absent	Mild	Moderate to big
Blood circulation	Regular and continuous flow	Irregular and/or decreased flow	Absent flow
Somites	V-shaped	C-shaped	Straight
Chorda	Smooth, straight and well demarcated border	Tortuous border with bulging	Tortuous and not well demarcated border

### Evaluation of cardiovascular effects

After performing microinjection as described above, structural cardiac defect and functional cardiac effects of the methylxanthine drugs were evaluated together with other cardiovascular endpoints.

### Structural cardiac defect

At 48 hpf, embryos were evaluated for any structural cardiac abnormality and categorized into two groups: normal phenotype, i.e. embryos with no visible cardiac abnormality, and embryos with cardiac phenotype, i.e. embryos with visible structural cardiac abnormality. Then, the percentage of embryos with cardiac phenotype was calculated for each of the drugs. Furthermore, the cardiac phenotype embryos were graded into '3', '2' and '1', based on the criteria modified from Panzica-Kelly et al., 2010 [34].

**Table:** 

Normal heart morphology with slightly smaller ventricle	3
Atrium and ventricle were either compressed or severely enlarged and misshaped with no clear boundary between them	2
Atrium and ventricle were severely deficient, misshaped and not well defined	1

The structural cardiac abnormality was identified under direct visualization of the heart of zebrafish embryos using Leica MZ 16F Stereomicroscope (Germany) at 80X magnification.

### Evaluation of cardiovascular endpoints

In addition to the structural cardiac defects, we also evaluated the presence of several cardiovascular endpoints which included pericardial edema, decreased or absent blood flow, arrhythmia, hemorrhage and thrombosis. The total number of embryos developing each of these endpoints with all the eight methylxanthines was documented and the resulting percentage calculated for each of the endpoints at 48 hpf.

To see the fate of the embryos with cardiac defects beyond 72 hpf, one selected concentration of each of the eight compounds was injected into the embryos at 1 cell stage. The embryos with cardiac defects were then evaluated for mortality till 5 dpf.

### Functional cardiac effect

To study the functional cardiac effects of methylxanthines, the heart rate of normal looking embryos was measured at 48 hpf. Normal looking embryos implied the embryos with intact structural integrity including the normal cardiac strucuture and cardiovascular endpoints. For each drug, ten embryos were randomly selected from a pool of normal looking embryos and the heart beat of each of the embryos was counted manually for 30 s under Leica MZ16F stereomicroscope (Germany) in quiet condition and at optimal temperature. The heart beat per 30 s was multiplied by 2 to obtain the heart beat per minute i.e. heart rate. Metoprolol, a beta blocker which decreases the heart rate in human, was used as an internal control and embryos injected with sterile water was used as negative control.

To further understand if the functional cardiac effect was reversible or irreversible, we performed the same experiment in the Tg(*Bmp:EGFP*) transgenic line with the embryos injected with one concentration of each drug. BMP transgenic line allows for the easy visualization of heartbeat, especially at 72 and 120 hpf when the pigmentation might make it difficult to count the heart beat in the wild type embryos. The heart rate was measured starting from 48 hpf till 120 hpf, at 24-h interval. The procedure for the measurement of heart beat was same as described earlier. However, for the measurement of heart beat at 72, 96 and 120 hpf, embryos were stabilized by briefly placing them for 10-15 s in low dose tricaine (0.16 mg/mL) before measuring the heart beat.

### Statistical analysis

All the experiments were repeated at least three times and the data are reported in Mean ± S.D. One way ANOVA with Dunnett’s test was used for the comparison of the heart rate of the treated embryos with control. Statistical analysis was done in graph pad prism 5 (La Jolla, CA USA) with *p* value <0.05 considered statistically significant.

## Results

### Embryotoxicity

As a first step in the characterization of the toxicity of methylxanthine compounds, we studied the lethality and phenotypic effects.

Five increasing doses/graded doses of eight methylxanthines: aminophylline, caffeine, diprophylline, doxofylline, etophylline, 3-isobutyl-1-methylxanthine (IBMX), pentoxifylline and theophylline; were injected into the yolk of 1-2 cell zebrafish embryos (Table 1) and the survival and phenotype analyzed at specific timepoints. The doses were selected in such a way that they fall within per oral therapeutic range in human. As the weight of zebrafish embryos is roughly 1.2 mg, administering 1 ng of a drug in one embryo (1 ng/1.2 mg) is approximately equal to 1 mg/kg of drug. Therefore, the doses of drugs used in microinjection were in the range of 1-20 mg/kg and most of these drugs have per oral therapeutic dose that falls within this range in humans.

**Fig. 1:**
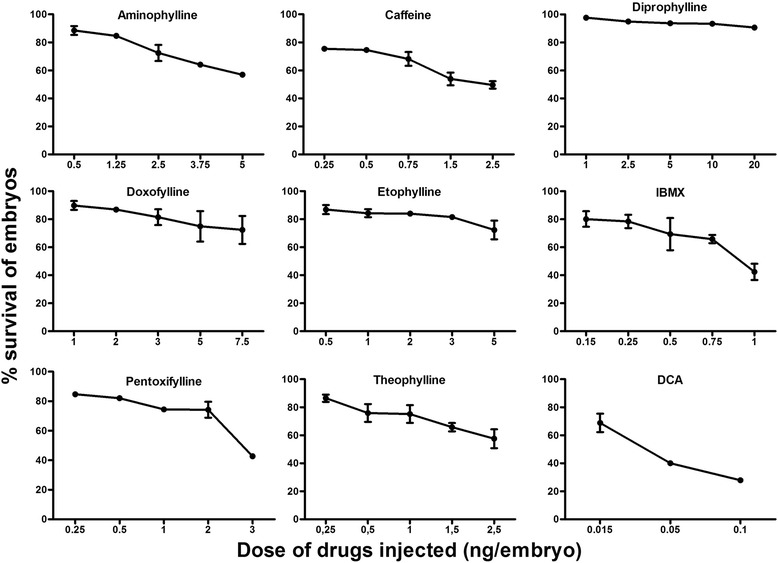
Dose-response curves depicting the survival of zebrafish embryos at 72 hpf after microinjection with eight methylxanthines at 1-2 cell stage. X-axis shows the amount of drug injected in the embryos; Y axis shows the corresponding survival percentage at 72 hpf. Embryos injected with dichloroaniline (DCA) were used as positive controls. At least 100 embryos were injected for each compound and the results are the mean ± SD of three independent experiments

Aminophylline, caffeine, doxofylline, IBMX, pentoxifylline and theophylline treated embryos showed dose dependent survival at 72 hpf, whereas the survival of diprophylline and etophylline treated embryos was independent of the doses (Fig. 1). IBMX was the most toxic/active compound: a large percentage of the embryos (58%) showed mortality when injected with 1 ng of the drug. Diprophylline was the least active methylxanthine; with more than 90% of embryos surviving at dose as high as 20 ng/embryo. Pentoxifylline and caffeine also produced >50% mortality, although they required higher doses than IBMX, i.e. 3 ng and 2.5 ng per embryo respectively to produce the equivalent mortality.

To simplify the comparison, we observed the dose at which there was a minimum of 20% mortality for each drug. Methylxanthines like IBMX, caffeine, pentoxifylline and theophylline were highly active with <1 ng needed to induce 20% mortaility. Aminophylline, etophylline and doxofylline were moderately active with 2.5-5 ng required to induce 20% mortality and diprophylline was a non toxic compound which didn’t induce 20% mortality even with the highest dose of 20 ng. To further compare the toxicity of each individual compound, we calculated the lethal dose at which there was 50% mortality (LD_50_) for each drug using linear interpolation and extrapolation. Our results showed that IBMX was the most toxic drug (LD50 = 0.91 ng) followed by caffeine (2.4 ng), pentoxifylline (2.76 ng), theophylline (3.4 ng) and aminophylline (6.15 ng), etophylline (9.8 ng),doxofylline (29.3 ng) and diprophylline (167.5 ng).

### General Teratogenicity and general morphological defects

After evaluating the mortality, the alive embryos were observed for morphological defects at 48 and 72 hpf. The embryos with morphological defects were identified and classified into mild and severe phenotype based on the presence of different morphological endpoints (Table 2). In particular, embryos with defective heart formation, irregular heartbeat, decreased blood circulation, mild pericardial and yolk sac edema were categorized as mild phenotype. Embryos with perturbed anterior-posterior axis development with reduced tail detachment and poor formation of somites, defective heart formation, irregular heartbeat, absent blood circulation, severe pericardial and yolk sac edema were categorized as severe phenotype.

**Fig. 2:**
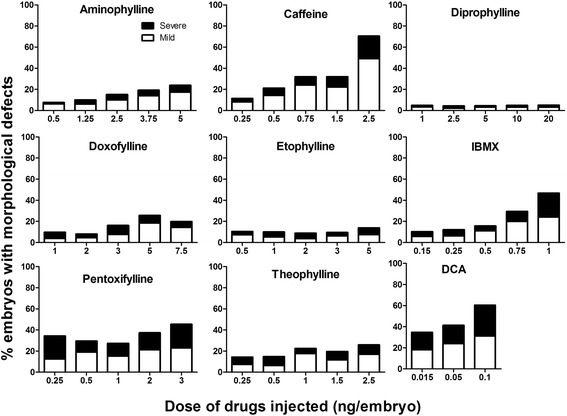
Percentage of embryos showing morphological defects at 72 hpf after microinjection with eight methylxanthines at 1-2 cell stage. For each drug, the percentage of embryos with mild (white bar) or severe (black bar) phenotype was assessed at each of the five tested concentrations. Embryos injected with dichloroaniline (DCA) were used as positive controls. At least 100 embryos were injected for each compound and the results are the mean ± SD of three independent experiments

**Fig. 3:**
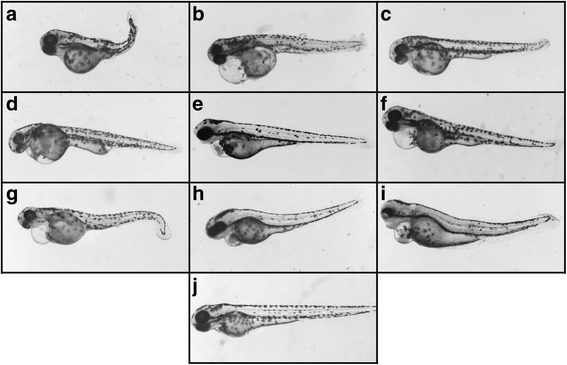
Representative pictures of zebrafish embryos at 72 hpf injected with: **a** Aminophylline; **b** Caffeine; **c** Diprophylline; **d** Doxofylline; **e** Etophylline; **f** IBMX; **g** Pentoxifylline; **h** Theophylline; **i** Dichloroaniline (positive control); and **j** Sterile water (negative control). The zebrafish embryos representative pictures were obtained with Leica MZ16F stereomicroscope(Germany) equipped with DFC 480 digital camera and LAS Leica Imaging software (Leica).Magnification 40X

The percentage of embryos that showed morphological defects with the highest concentration of drug varied with each compound. While the lowest dose used produced morphological defects in <10% of the embryos, the highest concentration produced morphological defects in 50% of embryos with 2.5 ng of caffeine, 44% with 1 ng of IBMX, 38% with 3 ng of pentoxifylline, 29% with 5 ng of aminophylline, 24% with 1 ng of theophylline, 23% with 7.5 ng of doxofylline, 15% with 5 ng of etophylline and 5% with 20 ng of diprophylline. The embryos injected with the positive control DCA showed 47% morphological defects, while less than 1% of negative controls injected with sterile water showed morphological defects (Fig. 2). These embryos predominantly had pericardial edema, abnormal blood circulation, yolk sac edema and abnormal AP axis formation (Fig. 3).

For the comparison of the morphological defects, we looked into the dose required to induce morphological defects in at least 20% of surviving embryos for each drug. The results showed that caffeine, IBMX, pentoxifyline and theophylline required ≤1 ng of drug; aminophylline and doxofylline required 3-5 ng of drug to induce the morphological defects in at least 20% of the embryos. However, diprophylline and etophylline didn’t cause morphological defects in 20% of the embryos even with the highest doses of 20 ng and 5 ng respectively.

### Cardiac toxicity and Teratogenicity

The cardiovascular effects of methylxanthines in zebrafish embryos were more extensively evaluated. Zebrafish embryos were injected with the same drugs and doses (see Table 1) at 1-2 cell stage. Embryos were then analyzed for the structural and functional alterations.

### Structural alteration

**Fig. 4:**
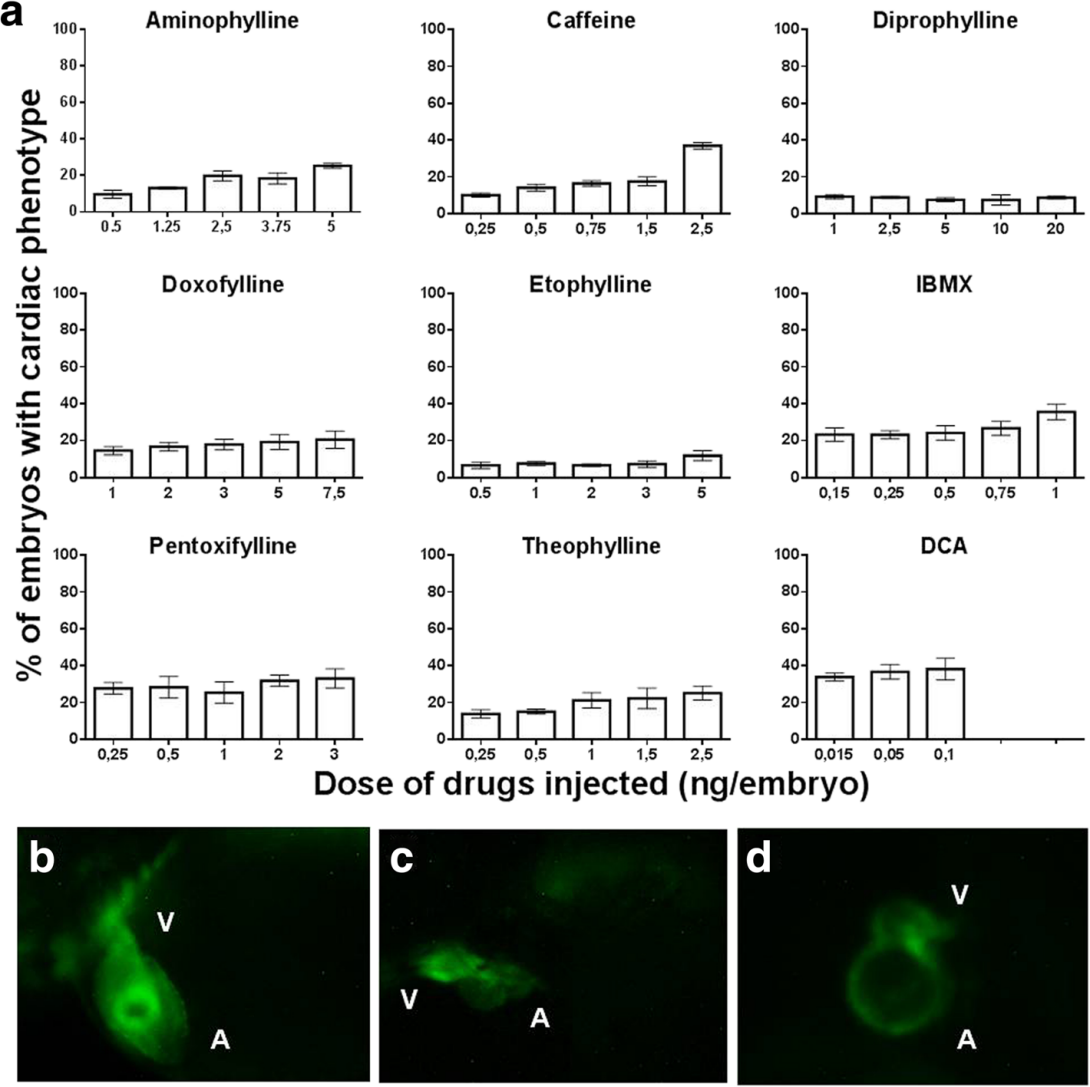
Structural alteration of the heart at 48 hpf. **a** Percentage of embryos with structural cardiac abnormality at 48 hpf after microinjection with eight methylxanthines at 1-2 cell stage. Embryos injected with DCA were the positive controls. At least 100 embryos were injected for each drug and the results are the mean ± SD of three independent experiments. **b** Representative picture of normal heart morphology from lateral view. **c** and (**d**) Representative pictures of grade 2 cardiac abnormality observed in methylxanthine treated zebrafish embryos at 48 hpf. Both atrium and ventricle are compressed and not well defined between chambers (**b**) or atrium and ventricle are severely enlarged, misshaped and not well defined (**c**). A- Atrium; V-Ventricle

Assessment of the structural alteration of the heart was performed at 48 hpf. The embryos showing structural cardiac defect were termed cardiac phenotype and embryos with normal cardiac morphology were termed normal phenotype. The total percentage of embryos with cardiac phenotype was calculated for each of the compounds (Fig. 4a). Each of the embryos with cardiac phenotype was further assessed and graded based on the the criteria modified by the study of Panzica Kelly et al. [34]. The structural cardiac deformity in the embryos was scored as follows: 3 in case of normal heart morphology with slightly smaller ventricle, 2 if the atrium and ventricle were either compressed or severely enlarged and misshaped with no clear boundary between the atrium and ventricle and 1 if the atrium and ventricles were severely deficient, misshaped and not well defined.

Our results showed that with the exception of diprophylline and etophylline, all the other drugs induced cardiac phenotype in 20-40% of embryos with the highest dose. The minimum dose that caused at least 10% cardiac defects was 0.15 ng for IBMX, 0.25 ng for caffeine, pentoxifylline and theophylline,1 ng for doxofylline, 2.5 ng for aminophylline and 5 ng for etophylline. With diprophylline, the cardiac defect was present in less than 10% of the embryos even with the highest concentration of 20 ng. Further analysis of the embryos with cardiac phenotype showed that most of them had grade 2 cardiac abnormality. They had severely enlarged atrium and ventricles with distorted shape and not clearly defined border (Fig. 4c, d).

### Evaluation of cardiovascular endpoints

In the embryos with structural cardiac defect, we also looked for several other cardiovascular endpoints at 48 hpf (Table 3). We observed that most of these embryos also had pericardial edema and decreased or absent blood circulation. Haemorrhage occurred only in few embryos injected with caffeine and theophylline, while thrombosis was absent in all the drug injected embryos.

To see the long term fate of the embryos with cardiovascular alterations, one concentration of each drug was injected in the BMP transgenic zebrafish embryos and tracked till 120 hpf. The embryos with structural cardiac defects either died or deteriorated further by 120 hpf.

**Table:** Table 3

		Cardiovascular endpoints				
Methylxanthine	Dose	Pericardial edema	Decreased blood flow	Absent blood flow	Thrombosis	Hemorrhage
Aminophylline	LC	2%	1%	1%	0	0
	HC	14%	16%	6%	0	2%
Caffeine	LC	0.01%	0.01%	0	0	0
	HC	12%	6.5%	9.8%	0	5.4%
Diprophylline	LC	0.01%	0.01%	0.01%	0	0
	HC	7.8%	4.95%	2.94%	0	0.01%
Doxofylline	LC	4%	4%	12%	0	0
	HC	13.7%	8.21%	11%	0	0
Etofylline	LC	0.8%	3%	2.5%	0	0
	HC	10.2%	5.6%	7.8%	0	0
IBMX	LC	3.8%	0	0.03%	0	0
	HC	18%	12.5%	15.3%	0	0.01%
Pentoxifylline	LC	0	0	0	0	0
	HC	8.5%	8.5%	5.7%	0	0
Theophylline	LC	0.01%	0.01%	0	0	0
	HC	17%	9.2%	13.8%	0	7.7%

Affected endpoints of cardiovascular system at 48 hpf. The table illustrate the percentage of embryos with a particular endpoint affected at the lowest and highest tested concentration

*LC* lowest concentration, *HC* highest concentration

### Functional alteration

Heart rate in the normal looking embryos was measured at 48 hpf to assess the functional alteration of cardiac system. As methylxanthines are adenosine receptor antagonist, we expected to observe an increase in heart rate in the treated embryos if they were affected at functional level. Ten embryos were randomly selected for each concentration of each drug and the heart beat was counted. Embryos treated in sterile water were used as a negative control and metoprolol treated embryos were used as an internal control.

**Fig. 5:**
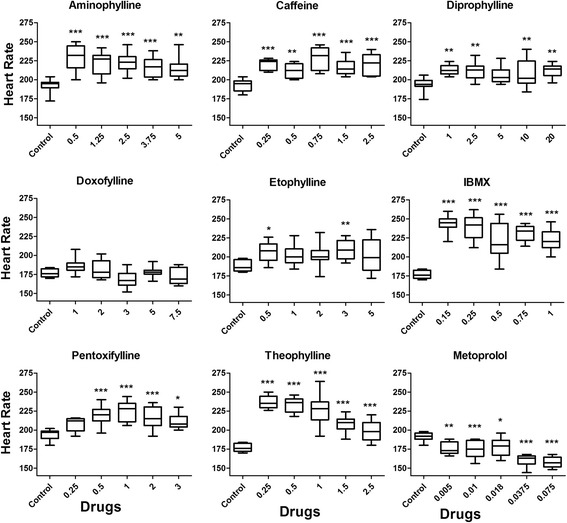
Heart rate of zebrafish embryos at 48 hpf. Ten normal embryos were randomly selected for each concentration of each drug for counting the heart beat. One way ANOVA with Dunnett’s test was used to test the significance of the data. Asterisks indicate that the *p*-value differ significantly from control. *** = *p* < 0.0005; ** = *p* < 0.005; * = *p* < 0.05

Our results showed that all the methylxanthines with the exception of doxofylline caused a significant increase in heart rate as compared to controls (Fig. 5). Doxofylline did not alter the heart rate whereas metoprolol significantly decreased the heart rate.

To see if the functional effect of these drugs in the embryos was a long lasting effect, the heart rate was tracked every 24 h from 48 hpf till 120 hpf in the BMP transgenic zebrafish embryos. We found that there was no significant difference in the heart rate in the treated and control embryos at 72, 96 and 120 hpf (Additional file 1).

## Discussion

### Embryotoxicity and general Teratogenicity

In the present study, we showed that if the exposure happens during the early phase of cellular differentiation, a single dose of methylxanthines might be sufficient to induce embryotoxicity and teratogenicity. Almost all the tested methylxanthines induced embryotoxic and teratogenic effects of varying intensity. Based on their potency, these drugs could be classified into three groups: highly toxic and teratogenic, toxic and teratogenic at higher doses and non toxic and teratogenic. Caffeine, IBMX, pentoxifylline and theophylline were highly toxic and teratogenic whereas aminophylline doxofylline and etophylline induced toxicity and teratogenicity but required higher doses. On the other hand, diprophylline induced minimal teratogenicity and toxicity which was comparable to that observed in control-injected embryos. The potency of these drugs in zebrafish embryos was also similar to their potency in human and other animal models. Indeed, studies on humans and other vertebrates have shown that etophylline, doxofylline and diprophylline have lesser side effects than other methylxanthines [12, 35]. Also, in vivo and in vitro studies have shown theophylline to be more potent than etophylline (7 fold more potent), diprophylline (100 fold more potent) [35] and doxofylline (10 fold more potent) [36]. Moreover drugs like doxophylline and diprophylline have fewer side effects as compared to other methylxanthines such as theophylline [10, 12]. Overall, our results showed that a single dose of these drugs could be embryotoxic and teratogenic in the zebrafish embryos with potencies comparable to the other higher animal models.

### Cardiac toxicity and Teratogenicity

The cardiac toxicity of the methylxanthines can be divided into structural and functional aspects. In our study, structural cardiac defect was not as profound as the functional effect. While the functional effect was present in all the drug treated embryos, structural cardiac defect was present in <40% of embryos at the highest concentration with all the methylxanthines. In particular, diprophylline and etophylline induced cardiac defect in less than 10% of embryos even at the highest injected concentration. Therefore, our results showed two facets of methylxanthine toxicity: structural and functional. While the structural alteration led to death, the functional alteration was transient and mild with the embryos ultimately recovering. One of the major adverse effects of methylxanthines is cardiac toxicity. In human, acute toxicity of methylxanthines cause functional alteration of cardiac physiology such as tachycardia and arrhythmia [1, 21] rather than the structural alteration of heart. However, animal studies have shown association of methylxanthines with structural cardiac defect [37, 38] and systematic review has shown cardiovascular malformations as a teratogenic effect of maternal caffeine ingestion in human [39]. Our study showed that methylxanthine induced structural alteration has lower incidence than the functional alteration. However, it could potentially be fatal. In this study, embryos were exposed to doses that fall within the therapeutic range in human. Although, interspecies differences need to be considered before drawing any conclusion, our study indicated that a single exposure of methylxanthines at therapeutic range could induce both functional and structural cardiac disfunction if exposed during early pregnancy before cellular differentiation.

### Comparison of cardiac effects with higher vertebrate studies

Human and animal studies have shown that most of the methylxanthines increases the heart rate [10, 13, 15, 35, 40, 41], whereas doxofylline does not have any effect on it [10, 36, 42, 43]. These findings were similar to the results we obtained in the zebrafish embryos where all the methylxanthines increased the heart rate and doxofylline did not have any effect. Therefore, at functional level, there was similarity in the effects of methylxanthines in zebrafish embryos and other animal models and human. The ability to respond to both the cardiac stimulant such as methylxanthines and depressant drug such as metoprolol also confirmed the functional maturity of the cardiac system of zebrafish embryos at 48 hpf, which is well supported by other zebrafish studies (mentioned in Review [20, 44]).

## Conclusion

We investigated the general and cardiac toxicity and teratogenicity of various methylxanthines: aminophylline caffeine, diprophylline, doxofylline, etophylline, IBMX, pentoxifylline and theophylline.

Our results showed that barring diprophylline, a single exposure of these drugs before cellular differentiation could lead to embryotoxicity and general teratogenicity together with the structural and functional alteration of the cardiovascular system. Of all the tested drugs, diprophylline appeared to be safer with fewer incidences of embryotoxicity, general and cardiac teratogenicity (< 10% of all injected embryos).

## Additional file

Additional file 1: Heart rate of BMP transgenic zebrafish embryos [tg(*Bmp:EGFP*)] at 48, 72, 96 and 120 hpf after microinjection with one dose of each drug. The doses selected were: aminophylline 2.5 ng, caffeine: 0.75 ng, diprophylline 5 ng, etophylline 3 ng, theophylline 2 ng, IBMX 0.5 ng, pentoxifylline 1 ng, theophylline 1 ng. For each drug, ten normal embryos were randomly selected for counting the heart beat. One way ANOVA with Dunnett’s test was used to test the significance. Asterisk indicates that the *p*-value differ significantly from control. (JPEG 1268 kb)

## Abbreviations

DCA: 3, 4-dichloroaniline
hpf: hours post fertilization
IBMX: 3-isobutyl-1-methylxanthine
LD: Lethal dose
ng: nanogram
