# Supplementary figures and images for: Methylxanthines induce structural and functional alterations of the cardiac system in zebrafish embryos
Source: BMC Pharmacol Toxicol. 2017 Nov 15;18:72. doi: 10.1186/s40360-017-0179-9 (PMC5688754; doi:10.1186/s40360-017-0179-9)

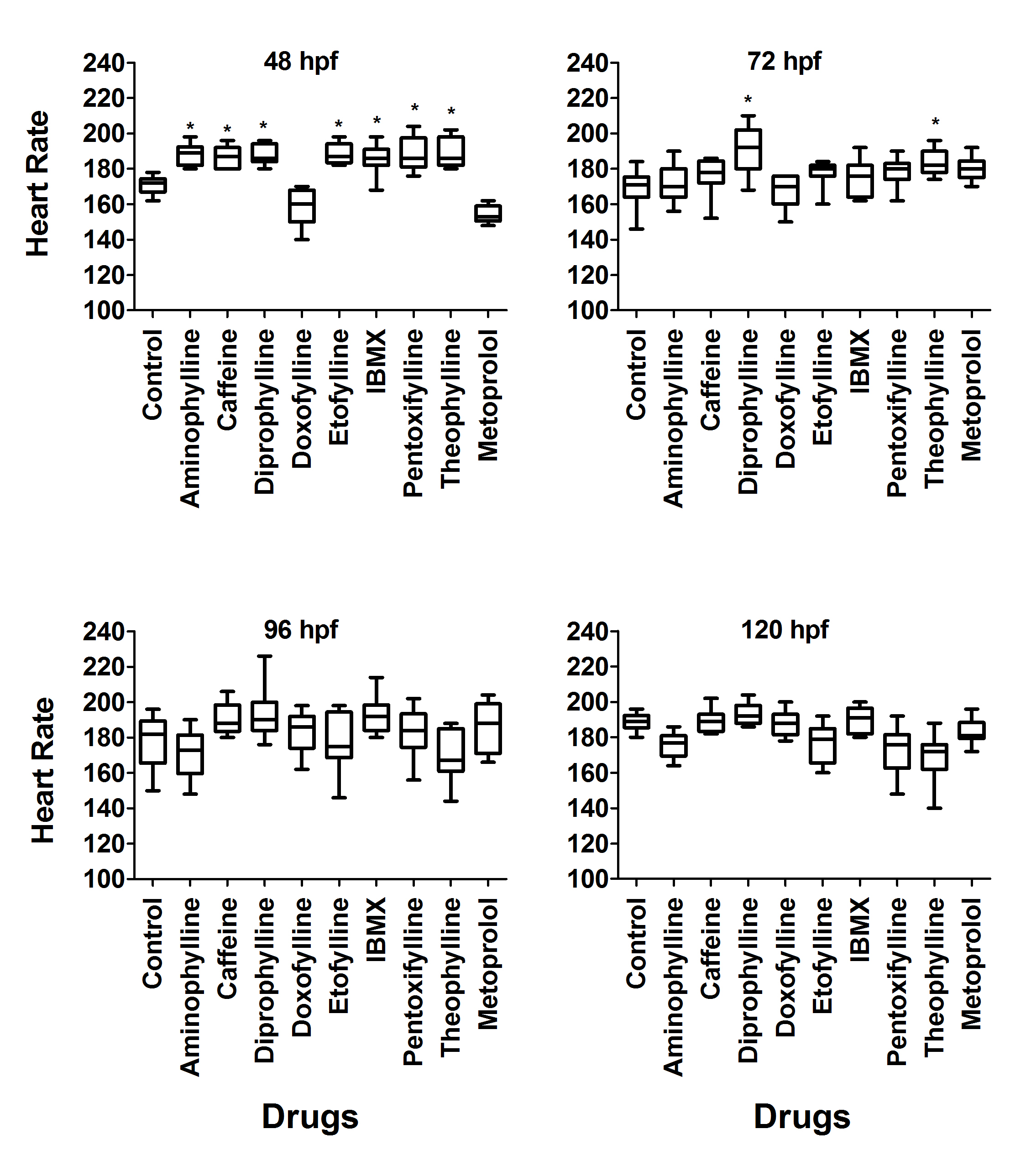

Supplement: Additional file 1: — Heart rate of BMP transgenic zebrafish embryos [tg(Bmp:EGFP)] at 48, 72, 96 and 120 hpf after microinjection with one dose of each drug. The doses selected were: aminophylline 2.5 ng, caffeine: 0.75 ng, diprophylline 5 ng, etophylline 3 ng, theophylline 2 ng, IBMX 0.5 ng, pentoxifylline 1 ng, theophylline 1 ng. For each drug, ten normal embryos were randomly selected for counting the heart beat. One way ANOVA with Dunnett’s test was used to test the significance. Asterisk indicates that the p-value differ significantly from control. (JPEG 1268 kb) [file 40360_2017_179_MOESM1_ESM.jpg]
